# Time-Dependent Effect of Oral Morphine Consumption on the Development of Cytotrophoblast and Syncytiotrophoblast Cells of the Placental Layers during the Three Different Periods of Pregnancy in Wistar Rats

**DOI:** 10.1155/2013/974205

**Published:** 2013-03-03

**Authors:** Leila Dehghani, Hedayat Sahraei, Rokhsareh Meamar, Masoomeh Kazemi

**Affiliations:** ^1^Department of Medical Sciences, Najafabad Branch, Islamic Azad University, Isfahan, Iran; ^2^Neuroscience Research Center, Baqiyatallah University of Medical Sciences, Tehran 1956837173, Iran; ^3^Isfahan Neurosciences Research Center, Isfahan University of Medical Science, Isfahan, Iran

## Abstract

Previous studies have shown that morphine abuse during pregnancy cancause a delay in the development of the placenta and embryo and also bring about birth defects. The present study investigates the effect of the duration of maternal morphine consumption during pregnancy, as well as the impacts of morphine abuse on the development of placental layers during the three different periods of pregnancy in Wistar rats. *Materials and Methodology*. Female Wistar rats have been used in the present study. Experimental groups received morphine (0.05 mg/mL of drinking water) after one night of coupling with male rats for mating. On 9th, 10th, and 14th days of pregnancy, pregnant animals were killed, and placentas were removed and fixed. The cells of the placentas layers were calculated by light microscope and MOTIC and SPSS software. *Results*. The maternal surface thickness of the placenta was significantly increased, whereasthe fetal surface thickness of placenta was significantly decreased with morphine consumption with a time-dependent manner in experimental groups, compared to control groups. Moreover, the number of trophoblast cells increased in both maternal and fetal surfaces of placenta with respect to the duration of morphine consumption which was overt in the experimental groups compared to the control groups. *Conclusion*. In general, the time-dependent effects of oral morphine consumption can inhibit the development and natural functioning of cytotrophoblast and syncytiotrophoblast cells of the placental layers.

## 1. Introduction

Opioid addiction is one of the major problems of the modern world. The harmful consequences of drug abuse do not only affect the addicted person. As an instance, some pregnant mothers abuse drugs and transmit the harmful effects of addiction to the future generations. The present study examines the effect of the duration of morphine addiction on the severity of developmental delay in the placental cytotrophoblast cells. Since the placenta is the main source of material transfer between the mother and the fetus, it is thoroughly exposed to the risks of addiction. The adverse effects of opioids in human subjects and in laboratory animals are well evident.

Experiments have shown that opioid drugs harm the addicted mother and also cause abnormalities in the development of the placenta and embryo. For example, drug abuse by pregnant mothers leads to a delay in the placental development and causes fetal defects such as spina bifida [1, 2]. In addition, many symptoms have been reported in the newborns of opioid-addicted mothers. According to some research, these symptoms may be due to a delay in the differentiation of the nervous system [2]. Also, since the placenta plays an essential role in fetal development, any kind of placental dysfunction leads to abnormalities in the growth and development of the embryo [3, 4].

Studies conducted in animal models have shown that morphine prescription during the embryonic period causes backwardness in the growth and development of the nervous system. For example, daily injection of morphine decreases locomotor activity in chickens. Administration of morphine also brings about a decrease in placental weight and the weights of most organs including the brain, liver, and kidney in rabbits [3]. Experiments have also indicated that morphine can cross the blood-placenta barrier, affect the embryonic cells, and cause contraction of the chorionic villi [2]. With the progress of gestation, the placenta forms into two portions: the maternal portion and the embryonic portion. The fetal portion of the placenta develops by the evolution of the villous chorion, and the maternal portion originates from the main decidua. In mammals, the placenta is the most important channel for the exchange of materials between the maternal and fetal bloods. On the other hand, the placental cells act as the major source of the secretion of the hormones required for the growth and development of the embryo, in such a way that during the primary days of gestation, the syncytiotrophoblast cells cause the corpus luteum to survive by the secretion of human chorionic gonadotropin (HCG) and make the embryo sustained in the uterine endometrium by the secretion of progesterone and estrogen ([5–7]). With the progress of gestation, the placental cells become capable of secreting progesterone and estrogen and other hormones necessary for the growth and development of the embryo, and hence, any kind of dysfunction in the secretory activities of the placental cells causes a delay in the growth and development of the fetus. The placenta functions as a protective barrier to prevent the entry or exit of some materials and is also usually considered as protective mechanism against the factors causing the abnormalities. Due to morphine cationic lipophilic property, it easily crosses the placental barrier and causes many defects on the embryonic and neural tube development and cerebellum as well [5, 6].

Based on the previous studies, morphine causes a delay in the development of neural tube, cerebellum, brain cavities, and the ependymal cells in the choroid plexus area of the nervous system [8–11]. Also, oral morphine consumption causes difficulties in the formation of placental blood pools in pregnant Wistar rats. According to the studies, prescription of oral morphine during the pregnancy period increases the concentration of blood plasma corticosterone, and this phenomenon, in turn, enhances the functioning of morphine in delaying the placental development [12, 13]. The present study investigates the time-dependent effect of the addiction duration of maternal morphine consumption during pregnancy, as well as the impacts of morphine abuse on the development of placental cells with 10, 9, and 14 days of age.

## 2. Materials and Methodology

Female Wistar rats with an average weight of 170 to 200 grams were used in this experimental research. The rats were kept in pair cages, under the ambient temperature (24 ± 1°C) and with natural photoperiods (12 hours light and 12 hours dark). During the experiment, the rats were given adequate food and water. The ethical standards established with laboratory animals were compiled (under the supervision and approval of the ethics committee of Baqiyatallah University of Medical Sciences).

In this study, morphine sulfate provided by the Iranian Temad Company was orally used. The rats were divided into three groups (six experimental groups and six control groups), with each group including twelve rats (*n* = 12). A total number of 36 healthy female rats [10, 11] were coupled with male rats for mating (one male rat for each two female rats). After ensuring the pregnancy (observing vaginal plugs and the presence of sperm in vaginal smears), the female rats were separated from the male rats in the next morning and held within the same paired groups. From then on (the 0th day of gestation), the experimental groups received a daily morphine dose of 0.05 milligrams per milliliter (5 mg morphine in 1000 mL of drinking tap water for six rats). 

The amount of morphine consumption was calculated for 10 mL of water per rat, but it was supposed that each rat would receive any amount of water they needed. All of the rats were anesthetized by chloroform on the 9th, 10th, and 14th days of pregnancy [14, 15]. The placenta and uterus were removed from the mother rats' bodies and put in a solution of 10% formalin for two weeks. After this phase, the placentas along with the uterine endometrium were put in the tissue processing equipment, ready for molding. The samples were put in paraffin for molding. Then the procedure of block sectioning was performed using microtome (made in Germany, FIST), and the serial longitudinal (sagital) sections with a thickness of 5 micrometers were prepared. The sections were then placed on glass slides and stained with hematoxylin-eosin (H&E) method. After staining and preparation, the slides were examined microscopically. The placentas of the experimental groups were compared to those of the control groups, in terms of the development of layer thickness (in maternal—fetal portions) and the number of cells (in maternal—fetal portions). The MOTIC software was used for tissue measurement, which consists of a microscope which is connected to a computer and a monitor through a software program. This software provides the possibility of scanning the slides and is capable of performing different measurements. The number of cells in each layer was counted and compared between the experimental groups and the control groups.

## 3. Statistical Analyses

Data were reported as mean ± SEM. Differences between group means were assessed by a one-way analysis of variance (ANOVA) and unpaired sample *t*-test using the SPSS/PC computer program (version 18.0). (a) *P* value of <0.01 and (b) *P* value of <0.05 were considered as significant.

## 4. Results

**Figure 1:**
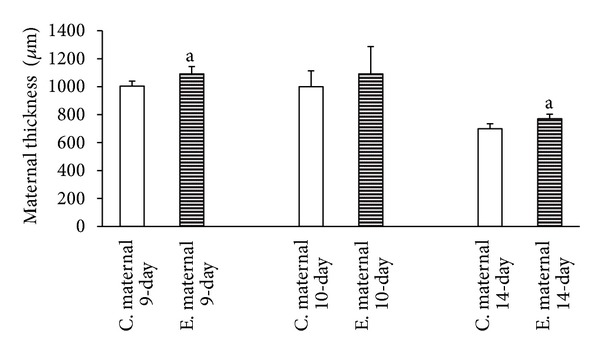
Reviewing the graph illustrating the time-dependent effect of oral morphine consumption on the development of the thickness of the maternal portion of the placenta for three different periods of pregnancy (9-day, 10-day, and 14-day pregnant mothers), and a comparison between the experimental (E) groups and the control (C) groups reveals the increase of the maternal portion of the placenta in the experimental group, compared to that of the control groups ((a) *P* value of <0.01 indicating a significant difference).

**Figure 2:**
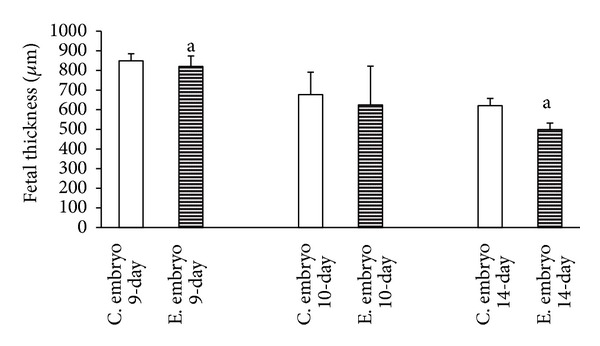
Reviewing the graph illustrating the time-dependent effect of oral morphine consumption on the development of the thickness of the fetal portion of the placenta for three different periods of pregnancy (9-day, 10-day, and 14-day pregnant mothers), and a comparison between the experimental (E) groups and the control (C) groups reveals the reduction of the fetal portion of the placenta in the experimental group, compared to that of the control groups ((a) *P* value of <0.01 indicating a significant difference).

**Figure 3:**
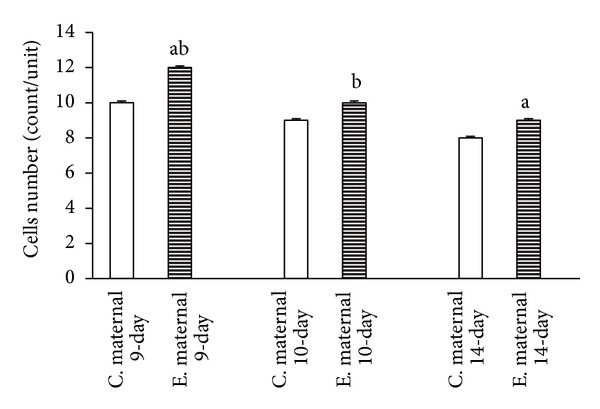
Reviewing the graph illustrating the time-dependent effect of oral morphine consumption on the development of the cells in the maternal portion of the placenta for three different periods of pregnancy (9-day, 10-day, and 14-day pregnant mothers), and a comparison between the experimental (E) groups and the control (C) groups reveals an increase in the number of cells in the maternal portion of the placenta in the experimental group, compared to that of the control groups ((a) *P* value of <0.01 and (b) *P* value of <0.05 indicating a significant difference).

**Figure 4:**
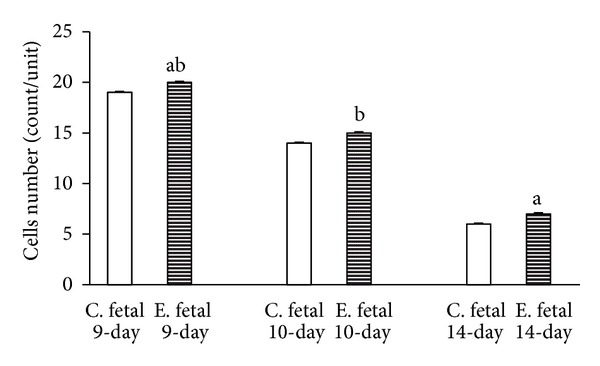
Reviewing the graph illustrating the time-dependent effect of oral morphine consumption on the development of the cells in the fetal portion of the placenta for three different periods of pregnancy (9-day, 10-day, and 14-day pregnant mothers), and a comparison between the experimental (E) groups and the control (C) groups reveals an increase in the number of cells in the fetal portion of the placenta in the experimental group, compared to that of the control groups ((a) *P* value of <0.01 and (b) *P* value of <0.05 indicating a significant difference).

The results of this study indicated that the time-dependent effect of the duration of maternal morphine consumption during different periods of pregnancy leads to an increase of thickness in the maternal portion of the placenta of the 9-day, 10-day, and 14-day groups. Thus, the thickness of the maternal portion of the placenta increases with morphine abuse in all three experimental groups compared to the control groups with a time-dependent manner. On the other hand, the findings of the present study, aligned with the body of research concerning the time-dependent effect of morphine consumption, revealed a significant reduction in the thickness of the fetal portion of the placenta in the experimental groups (Figure 1). This shrinkage of thickness as a time-dependent effect of morphine consumption was more evident in the placentas of 14-day pregnant mothers (Figure 2). Concerning the time-dependent effect of morphine consumption in pregnant mothers in terms of counting the number of cells in the maternal and fetal portions of the placentas in the experimental and control groups, a significant increase in the number of cells in both the maternal and fetal portions of the placenta was evident in the 9-day, 10-day, and 14-day pregnant subjects of the experimental groups, compared to subject of the control groups (Figures 3 and 4).

### 4.1. Morphological Observations and Measurements

**Figure 5:**
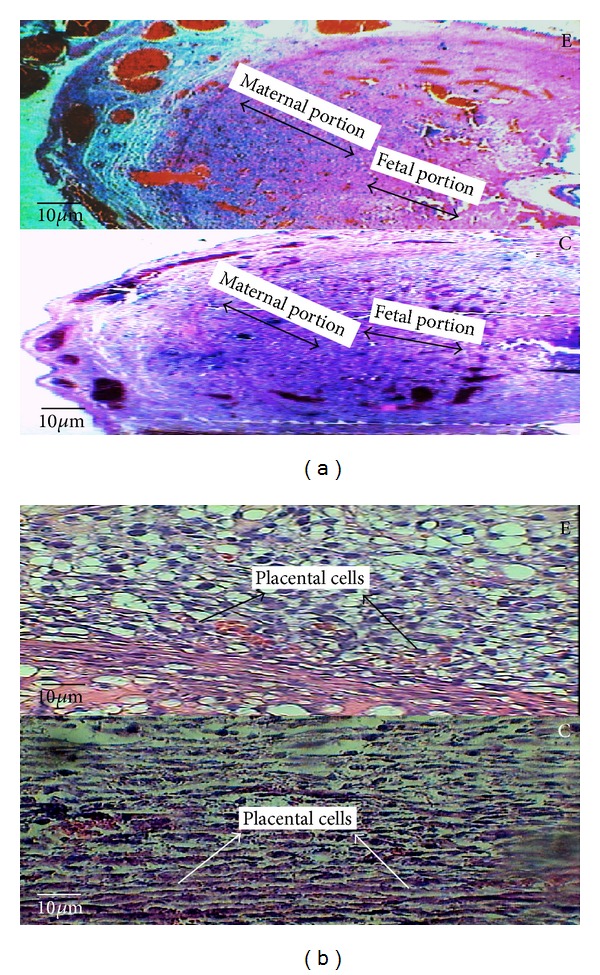
The microscopic image of the time-dependent effect of oral morphine consumption in the experimental (E) groups and the control (C) groups in the 9-day-old placenta with 40x magnification, (a) illustrating the thicknesses of both maternal and fetal portions of the placenta by the double-headed arrow. (b) The microscopic image of the experimental group and the control group in the 9-day old placenta with 40x magnification, illustrating the placental cells.

**Figure 6:**
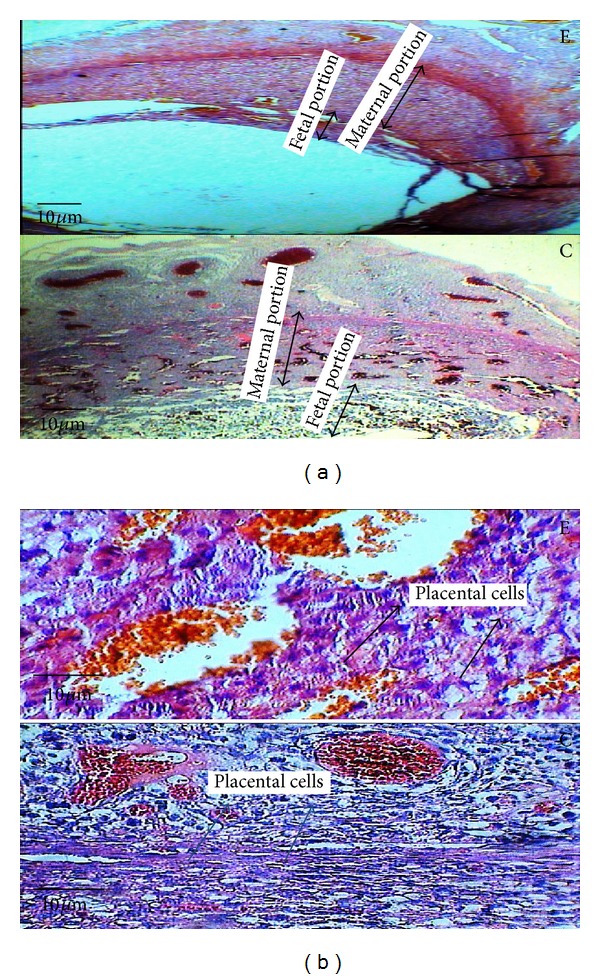
The microscopic image of the time-dependent effect of oral morphine consumption in the experimental (E) groups and the control (C) groups in the 10-day-old placenta with 40x magnification, (a) illustrating the thicknesses of both maternal and fetal portions of the placenta by the double-headed arrow. (b) The microscopic image of the experimental group and the control group in the 10-day-old placenta with 40x magnification, illustrating the placental cells.

**Figure 7:**
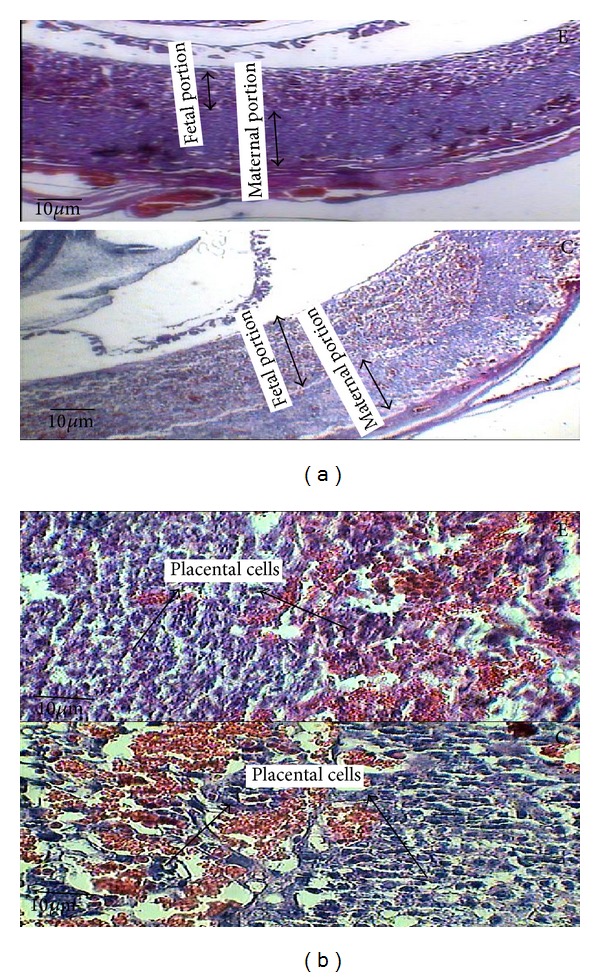
The microscopic image of the time-dependent effect of oral morphine consumption in the experimental (E) groups and the control (C) groups in the 14-day-old placenta with 40x magnification, (a) illustrating the thicknesses of both maternal and fetal portions of the placenta by the double-headed arrow. (b) The microscopic image of the experimental group and the control group in the 14-day-old placenta with 40x magnification, illustrating the placental cells.

The morphometric measurements showed that the time-dependent effect of oral morphine consumption leads to an increased thickness in the maternal portion of the placenta and a decreased thickness in the fetal portion of it in the experimental groups (Figures 5(a), 6(a), 7(a) in experimental and control groups). Also in the experimental groups, the number of cells in both the maternal and the fetal portions of the placenta was larger than that of the control groups (Figures 5(b), 6(b), 7(b) in experimental and control groups).

## 5. Discussion 

The present study, conducted to investigate the time-dependent effects of oral morphine consumption on the pregnant mothers during the three different (9-day, 10-day, and 14-day) periods of pregnancy, showed that the harmful consequences of oral morphine consumption vary with time. In fact, the observations of this study, suggesting the delaying effect of morphine in placental development, are in accordance with the findings of several studies, suggesting the impacts of opioid prescription in the induction of delayed differentiation [1, 2, 10, 11]. The importance of the results of this research lies in revealing the fact that prescription of oral morphine in the first half of gestation (9- and 10-day-old placentas) results in impacts different than the same prescription for the second half of gestation (14-day-old placenta) (Figures 1, 2, 3, and 4).

Today, it is known that the embryo is more sensitive to exogenous substances (such as drugs) in certain periods of pregnancy [4]. According to scientists' studies, morphine acts as a mitogenic stimulus. Morphine prescription also causes the release of stress hormones such as corticosterone. The researches have shown that the concentration of corticosterone in maternal serum increases during the pregnancy period ([1, 10, 11]). Previous studies have confirmed that morphine prescription causes an increase in the concentration of corticosterone, and this phenomenon, in turn, intensifies the functioning of morphine [13, 16]. The activity of corticosterone in the presence of morphine causes an increase in the blood pressure and hyperemia in the choroid plexus in rats. Scholars have stated that an increase in glucocorticoids weakens the placenta and the fetus. This phenomenon occurs directly with the change of cell cycle from the proliferation phase through the differentiation phase. Moreover, the opioids and corticosterone cause the cytotrophoblast cells to proliferate [1, 17, 18].

The results of the present study are consistent with the results of the previous researches in this field. This study suggests an increase in the thickness of the maternal portion of the placenta and a reduction in the thickness of its fetal portion as a result of oral morphine consumption during gestation, noting that the effect of intensity of morphine abuse during the first half of pregnancy was higher than in the second half. Thus, it is inferred that morphine acts as a mitogenic stimulus for cytotrophoblast cells and consequently thickens the maternal portion of the placenta (Figure 5). On the other hand, as mentioned before, prescription of oral morphine increases the concentration of blood plasma corticosterone. The corticosteroid hormone induces the mitosis of cytotrophoblast cells into syncytiotrophoblast cells, by shortening the interphase cell cycle. The shortening of the interphase cell cycle means an intervention in the natural mechanism of mitosis, during which the cells would not have enough time for protein synthesis, duplication of chromosomes, and natural growth and proliferation [12, 17].

The findings of this research, studying the time-dependent effects of oral morphine prescription and consumption during the pregnancy period, revealed a reduction in the thickness of the fetal portion of the placenta (particularly in the second half of gestation and for the 14-day old placenta), despite the increase in the number of cells in the same portion. This delay can be justified as follows: administration of morphine increases the concentration of corticosterone. Both the morphine and corticosterone mutually intensify the functioning of each other in a synergic manner and induce an increase in the mitosis of cytotrophoblast cells by shortening the interphase cell cycle for cytotrophoblast cells. Thus, the total number of cells is increased, but the growth and development are abnormal. The result is a reduction in the fetal portion, which is a time-dependent effect of oral morphine consumption and more evident in the 14-day old placenta (Figures 2 and 4).

 Since the fetal portion of the placenta is the most important channel for the exchange of materials between the mother and embryo, it encompasses the most blood vessels (blood pools) and the most opioid receptors on the endothelial cell membranes of chorionic villi [17, 18]. Every factor (morphine or corticosterone) that triggers cell proliferation without the natural growth, in fact, causes a delay in the normal development of the cytotrophoblast cells. This defect in the normal development of placenta leads to either abnormal fetal development or miscarriage [12, 16]. According to the conducted studies, administration of morphine to pregnant rabbits causes miscarriages and low birth weights in the newborns [3].

The main function of the placental cells is to secrete the hormones required for fetal growth and development, such as estrogen and progesterone. Indeed, pregnancy maintenance and fetal viability depend largely on the natural secretion of estrogen and progesterone hormones [4, 17]. 

Overall, these results show that most of the negative effects of oral morphine consumption by pregnant women are time-dependent, and the severity of the delay in the development of the placental layers, depending on the duration of morphine consumption, is more evident. In a general conclusion, it can be stated that the time-dependent effects of oral morphine consumption can cause a delay in the natural development of cytotrophoblast and syncytiotrophoblast cells of the placental layers in rats. The consequence of this delay is the defects and abnormalities in the natural formation of placental cells. This delay might be the main cause of disorders in the growth and development of newborns and miscarriages in addicted pregnant women [16]. However, further studies are required to disentangle this issue.

## 6. Conclusion 

Morphine causes abnormal proliferation of cytotrophoblast cells following changes in the thickness of fetal and maternal portions. According to the present study, the time-dependent effect of oral morphine consumption was shown as a result of significant increment in the thickness of maternal portions in placenta on the 9-10th and 14th days of pregnancy in comparison with control groups. This result may also be true for humans although behavioral disorders in infants or embryonic abortion from addictive pregnant mothers need to be further studied.

## Abbreviations

CP: Choroid plexus
H&E: Hematoxylin and eosin.
